# Cancer incidence increasing globally: The role of relaxed natural selection

**DOI:** 10.1111/eva.12523

**Published:** 2017-08-24

**Authors:** Wenpeng You, Maciej Henneberg

**Affiliations:** ^1^ Biological Anthropology and Comparative Anatomy Unit Adelaide Medical School The University of Adelaide Adelaide SA Australia; ^2^ Institute of Evolutionary Medicine University of Zurich Zurich Switzerland

**Keywords:** biological state index, cancer heritability, life expectancy, mutations

## Abstract

Cancer incidence increase has multiple aetiologies. Mutant alleles accumulation in populations may be one of them due to strong heritability of many cancers. The opportunity for the operation of natural selection has decreased in the past ~150 years because of reduction in mortality and fertility. Mutation‐selection balance may have been disturbed in this process and genes providing background for some cancers may have been accumulating in human gene pools. Worldwide, based on the WHO statistics for 173 countries the index of the opportunity for selection is strongly inversely correlated with cancer incidence in peoples aged 0–49 years and in people of all ages. This relationship remains significant when gross domestic product per capita (GDP), life expectancy of older people (*e*_50_), obesity, physical inactivity, smoking and urbanization are kept statistically constant for fifteen (15) of twenty‐seven (27) individual cancers incidence rates. Twelve (12) cancers which are not correlated with relaxed natural selection after considering the six potential confounders are largely attributable to external causes like viruses and toxins. Ratios of the average cancer incidence rates of the 10 countries with lowest opportunities for selection to the average cancer incidence rates of the 10 countries with highest opportunities for selection are 2.3 (all cancers at all ages), 2.4 (all cancers in 0–49 years age group), 5.7 (average ratios of strongly genetically based cancers) and 2.1 (average ratios of cancers with less genetic background).

## 1 INTRODUCTION

Worldwide, cancer incidence rate has increased to make it the second leading cause of death after cardiovascular disease. Environmental factors, such as tobacco smoking, urbanization and its associated pollution and changing diet patterns together with increased wealth associated with better medical services and extended postreproductive life span, have been considered responsible for this phenomenon. Prevention and treatment measures focusing on environmental factors have been implemented, but little progress in reducing incidence of cancers has been made (Global Burden of Disease Cancer Collaboration, 2015).

Malignant neoplasms are results of somatic mutations of certain genes (Croce, 2008; Vogelstein & Kinzler, 2004). Studies investigating transmission of cancer susceptibility in family lines suggested genetic background for incidence of many types of malignancies (Tian et al., 2015). It is possible, then that this background contributes to increasing incidence of cancers at the population level.

Mutations are more common than previously thought (Conrad et al., 2011; Crow, 2000; Henn, Botigué, Bustamante, Clark, & Gravel, 2015). For instance, it has been estimated that an average neonate has some 74 de novo point mutations (Conrad et al., 2011; Lynch, 2016). Multiple mutations may accumulate in genomes over time spanning just a few generations (Stephan & Henneberg, 2001). When selection against a certain mutation does not operate, the frequency of mutated alleles doubles every generation (Bodmer & Cavalli‐Sforza, 1976). The mutation load is directly proportional to the mutation rate and inversely proportional to the rate of selection (Bodmer & Cavalli‐Sforza, 1976; Crow, 1958). Thus, when selection rates approach zero, mutation load approaches infinity. These rates are expressed per generation. Human generations do overlap due to the length of the reproductive life span which in females is approximately 30 years. Assuming, for simplicity's sake, zero selection, it can be shown that mutation load at a given locus can triple or quadruple during one century (three to four generations). In the recent past, selection operating in human populations has been significantly relaxed (Crow, 1958; Lynch, 2016; Rühli & Henneberg, 2013) by medical and public health actions. This results in accumulation of mutations, especially mildly deleterious mutations. Interactions between alleles of various loci may magnify mutation rates including rates of somatic mutations that result in neoplastic cell growth because of the way DNA replicates and is repaired which is similar in germline and in somatic cells (Lynch, 2016). Combination of effects of mutations with relaxed selection produces a real possibility of deterioration of biological integrity of human organisms, observable in the time of a few generations in most advanced societies.

Human morphological characteristics that have a heritable, polygenic background have been evolving during the Holocene very fast; for example, rate of cranial capacity change was −10.8 darwins while the cranial index (the ratio of braincase width to its length) changed at a rate of +65.2 darwins (Rühli & Henneberg, 2016) and stature at +606.2 darwins (Henneberg, 2004). These are polygenic characters with incomplete heritability, and we cite them here as an illustration of how development of technological and social adaptations lowering natural selection rates in the last few millennia can influence the course of change in human biological characteristics.

Natural selection is a process that differentiates reproduction of individual genes into new generations depending on how genetic endowment of parents influences the number of offspring that will replace them in the future (Fisher, 1999). Following Fisher's (1958) definition of the reproductive value, “Biological State Index (*I*_bs_)” has been proposed to measure an opportunity for an average member of a population to pass genes to the next generation. *I*_bs_ calculation combines data on mortality and fertility (Budnik & Henneberg, 2017; Henneberg, 1976; Henneberg & Piontek, 1975; Stephan & Henneberg, 2001; You & Henneberg, 2016). The formula (Henneberg, 1976; Henneberg & Piontek, 1975) for *I*_bs_ calculation is as follows:

$$I_{bs}=1-\sum_{x=0}^{x=\omega }\;d_{x}s_{x}$$

where *d*_x_ = the frequency of deaths at age *x*;* s*_x_ = the probability of not possessing the complete number of births at age *x*;* ω* = the age at death of the oldest member of the group.

I_bs_ expresses a probability for an average individual born into a population to pass on genes to the next generation. Index value of 1.0 means that there is no opportunity for natural selection through differential mortality because all individuals survive until the end of their reproductive period.

This index is a more precise calculation than what Crow (1958) called the *P*_d_ (Crow, 1958)—proportion of individuals dying before reaching age of reproduction that is used to calculate the index of total selection due to mortality. For this index, a “…source of error is that no allowance was made for women who died during the childbearing period after having one or more children.” (Crow, 1958). In the *I*_bs,_ such allowance is made using *s*_x_ and *d*_x_ values for ages 15–49 years. By analogy to the Crow's mortality index of *P*_d_/*P*_s_ (where *P*_s_ is a proportion of individuals surviving to the reproductive age), an index of total opportunity for selection through differential mortality (including its portion during reproductive years) is constructed *I*_s_ = (1‐*I*_bs_)/*I*_bs_. Theoretically, following Fisher's formulation, the opportunity for selection must include the variance of fertility, more precisely, this portion of the variance of fertility that is heritable *V*_f_/*x* ^2^ (where *x* is the average number of children per female surviving to the menopause and *V*_f_ variance of this number). In humans, however, heritable variance in actual fertility is very low even in couples who do not control family size. According to our study (Henneberg, 1980) of 7,503 births from 1,525 Polish and American historical couples in 12 groups free of conscious birth control, the genetic variance of fertility is less than 0.01 of its squared mean. Furthermore, considering that in developed countries, conscious birth control has been practiced for over 100 years and became widespread in at least the last two generations, further diminishing any heritable fertility differentials, the contribution of genetic variance in fertility to the opportunity for natural selection in humans is practically nonexistent. Therefore, the use of *I*_s_ measuring opportunity for selection through differential mortality provides sufficient approximation of the maximum selective pressures in modern human populations.

The primary role of natural selection is that of the “janitor of the gene pool” purging deleterious mutations. In the past ~100 years, there has been a great reduction in mortality and in fertility that has been limiting the overall opportunity for natural selection (Henneberg, 1976; Saniotis & Henneberg, 2011; Stephan & Henneberg, 2001). It follows that genes potentially providing background for some cancers have been accumulating in various populations. Cancer incidence may be greater in those populations who have experienced less opportunity for natural selection.

We hypothesize that in a global perspective, extent of relaxation of natural selection in various national populations may be positively correlated with greater cancer incidence.

## 2 MATERIALS AND METHODS

The country‐specific variables were collected for this ecological study.

1. Dependent variables: The GLOBOCAN 2012 estimates of incidence rates (C50) (Ferlay et al., 2013)

We extracted the cumulative incidence rates of all cancers excl. nonmelanoma skin cancer (C00‐97, but C44) among people of all ages and people aged 0–49 years, respectively, for 184 countries. We also captured separate estimates of incidence rates of 27 site cancers from the same source of data for people of all ages. The site cancers are as follows: lip and oral cavity (C00‐08), nasopharynx (C11), other pharynx (C09‐10,C12‐14), oesophagus (C15), stomach (C16), colorectum (C18‐21), liver (C22), gallbladder (C23‐24), pancreas (C25), larynx (C32), lung (C33‐34), melanoma of skin (C43), Kaposi sarcoma (C46), breast (C50), cervix uteri (C53), corpus uteri (C54), ovary (C56), prostate (C61), testis (C62), kidney (C64‐66), bladder (C67), brain (C70‐72), thyroid (C73), Hodgkin lymphoma (C81), non‐Hodgkin lymphoma (C82‐85,C96), multiple myeloma (C88 + C90) and leukaemia (C91‐95). The cancer incidence rate indicates the number per 100,000 persons who were diagnosed with cancer in 2012. The rate was age‐standardized using the world standard population to increase the comparability.

Women age 50+ years enter menopause, which brings their fertility to zero. Female reproductive behaviour has been associated with various female cancers (Britt & Short, 2012; MacMahon et al., 1970; Ramazzini, 1983). The *I*_bs_ reflects mortality up to the age 50 years, considered the end of the reproductive life span, because *s*_50+_ values equal zero (thus any *d*_50+_ values are multiplied by zero). This means that natural selection we measure cannot “reach” beyond age 50 years. For these reasons, we included specifically the incidence rate of all cancers in the age range 0–49 years (prereproductive and reproductive life span) as these cancers can directly produce mortality and fertility differentials influencing reproductive success of individuals.

1. Independent variable: The index (*I*_s_ = (1‐*I*_bs_)/*I*_bs_) of natural selection opportunity at population level

The *I*_bs_ was calculated (Henneberg, 1976; Henneberg & Piontek, 1975) with the data of the world fertility published by United Nations in 2008 (The United Nations, 2012) and the data of life tables published by World Health Organization (WHO) in 2009 (WHO, 2012).

James Crow (1958), based on the Fisher's (1958) concept of the reproductive value (Grafen, 2006), proposed to measure the total opportunity for natural selection (*I*) as the ratio of variance in offspring size of a couple (*V*) to the squared average offspring size of a couple (*x* ^2^) that will replace parents in the next generation. In the application to human populations, this approach encounters two problems. The first is the birth control, which is very substantial in many modern societies. The second one is the overlapping of generations due to long reproductive period of females and males. The first problem can be tackled by separating contributions of fertility and mortality to the opportunity for selection and using only the portion of selection resulting from mortality. According to Crow (Crow, 1958), the index of opportunity for natural selection through differential mortality (*I*_m_) is the ratio of individuals dying before reaching reproductive age (*P*_d_) to the individuals surviving (*P*_s_): *I*_m_ = *P*_d_/*P*_s_. As not all individuals surviving to reproductive maturity will survive through the entire reproductive life span, a correction for deaths during the reproductive period is needed. This is introduced in the form of the Biological State Index (*I*_bs_) that combines age‐specific mortality (*d*_x_) with age‐specific opportunity for producing offspring in the future life (*s*_x_) (Henneberg, 1976; Henneberg & Piontek, 1975). The Biological State Index accumulates mortality data in the way similar to “survival” biometric function of the life table and depends on the distribution of age‐specific relative fertility expressed as the fraction of the total fertility rate remaining to be produced by a person of age *x*. Multiplication of the *I*_bs_ value for a given population by the total fertility rate of this population (number of children born by females surviving to the menopause) produces the net reproductive rate, a generational measure of population growth. Details regarding *I*_bs_ are explained in several previously published studies (Budnik & Henneberg, 2017; Henneberg, 1976; Henneberg & Piontek, 1975; Rühli & Henneberg, 2013, 2016; You & Henneberg, 2016). Considering low heritable variance of fertility and the widespread birth control that allow us to neglect opportunity for natural selection through differential fertility (Henneberg 1980), the index of the total opportunity for selection in modern populations is *I*_s_ = (1‐*I*_bs_)/*I*_bs_. The lower the value of this index, the less opportunity for natural selection exists. None of the three indices discussed here (*I*_m_, *I*_bs_ or *I*_s_) has any unit because they are ratios of offspring numbers or proportions and probabilities. Indices of the opportunity for selection measure the upper limit of the total selection pressure. Actual selection pressures can be lower because not all mortality differentials are heritable, but the magnitude of selection cannot exceed index values. Therefore, decreasing values of opportunity for selection indices certainly show reduction in possibility of selection to occur, while they do not measure the actual magnitude of selection that can be lower.

**Figure 1:**
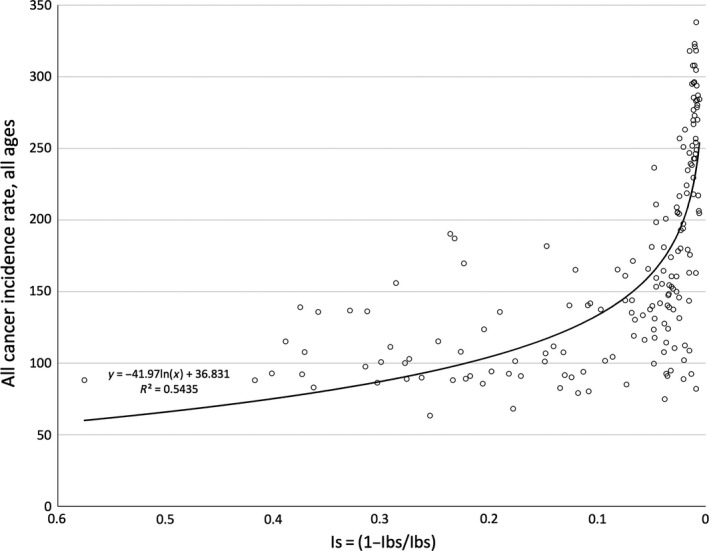
The relationship between *I*_s_ and all cancer incidence rate (all ages)

Gross domestic product per capita (GDP), life expectancy of older people (*e*_50_), obesity (BMI ≥ 30 kg/m^2^), prevalence rate (WHO, 2015), physical inactivity prevalence rate (Allender, Foster, Hutchinson, & Arambepola, 2008; Moore, Gould, & Keary, 2003; WHO, 2010), smoking and urbanization (Allender et al., 2008; Blot, Fraumeni, & Stone, 1977; Greenberg, 1983; Nasca, Mahoney, & Wolfgang, 1992) have been associated with cancer initiation. They were considered as the confounders when we conducted the data analysis in this study.

1. The World Bank published data (The World Bank Group, 2016) on GDP

GDP is used as the index of socio‐economic level, and it is expressed in per capita purchasing power parity (PPP in current international USD) in 2010. Socio‐economic levels measured with GDP have been related to cancer incidence rate (Blot et al., 1977; Ferlay et al., 2013, 2015; Jemal et al., 2011).

1. The United Nations Statistics Division estimates of the life expectancy (United Nations‐Department of Economic and Social Affairs‐Population Division, 2013)

Increasing life expectancy of older people, indexing ageing in this study, has been considered as a factor possibly promoting increasing cancer incidence (Breastcancer.org, 2016; Majeed, Babb, Jones, & Quinn, 2000). Therefore, the life expectancy (*e*_50_, 1990–1995) was extracted from abridged life tables (1950–2100) (United Nations‐Department of Economic and Social Affairs‐Population Division, 2013) published online by the United Nations.

1. The WHO Global Health Observatory (GHO) data on estimated obesity prevalence rate, physical inactivity, smoking rate and urbanization (WHO, 2015)

The obesity prevalence is expressed as the per cent of population (2010) aged 18+ with body mass index (BMI) ≥ 30 kg/m^2^.

Physical inactivity is defined as the per cent of a particular population attaining less than 150 min of moderate‐intensity physical activity per week, or less than 75 min of vigorous‐intensity physical activity per week, or equivalent in 2010.

Smoking is expressed as the per cent of adults aged 15 years and over (age‐standardized rate) who smoked any tobacco product daily in 2010.

Urbanization is expressed with the per cent of total population living in urban areas in 2010. Urbanization, representing a major demographic shift, entails lifestyle changes, including diet with more energy dense components, such as high fat and high alcohol consumption in daily diet, and less physical exercise.

### 2.1 Data selection

We used country‐specific cancer incidence rates, life tables and fertility rates (for *I*_s_ calculation), GDP, life expectancy at 50 years of age (*e*_50_), obesity prevalence rate, physical inactivity prevalence rate, smoking and urbanization for all countries where data were available. We aligned cancer incidence rates with *I*_s_ by country, and we obtained a set of data consisting of 173 countries. Quality of the country‐specific cancer estimation depends upon the quality and the amount of the information available for each country (International Agency for Research on Cancer (IARC), 2012). For data robustness check, we clustered the countries with “high‐quality” data as defined by the International Agency for Research on Cancer (IARC) (International Agency for Research on Cancer (IARC), 2012),obtaining a subset of data comprising 64 countries. This smaller data set was analysed separately from the other set of data consisting of all 173 countries. Country‐specific GDP, life expectancy (*e*_50_), obesity prevalence rate, physical inactivity prevalence rate, smoking and urbanization were matched with the listing of 173 countries which have both cancer incidence rate and *I*_s_. Numbers of countries included in the analysis of relationships with other variables may have differed somewhat because all information was not uniformly available for all countries.

All data included in this study were published by UN agencies. No ethical approval or written informed consent for participation was required.

### 2.2 Data analysis

Various statistical analysis methods were applied in this study to explore the correlation between *I*_s_ and cancer incidence rates. Each country was treated as an individual subject in the analysis. To examine the correlation between *I*_s_ and cancer incidence rates, the analysis proceeded in five steps:

1. Pearson's *r* and nonparametric correlations (Spearman's “rho”) were used to evaluate the strength and direction of the correlation between all the variables. Pearson's correlations and partial correlations were calculated using log‐transformed (ln) variables to minimize nonhomoscedascity of their distributions. Fisher's z‐transformation of correlation coefficients was used to assess significance of individual correlation coefficients values and of differences between correlation coefficients values.
2. The independent relationships between *I*_s_ and each cancer incidence rate for all ages were explored with partial correlation of Pearson's moment‐product approach while we controlled for the six variables, which are GDP (Ferlay et al., 2015), life expectancy (*e*_50_) (American Cancer Society, 2015; Colditz & Wei, 2012; McPherson, Steel, & Dixon, 2000), obesity prevalence rate (Danaei et al., 2005; Colditz & Wei, 2012), physical inactivity prevalence rate (Danaei et al., 2005), smoking (Colditz & Wei, 2012; Danaei et al., 2005) and urbanization (Danaei et al., 2005). Life expectancy (*e*_50_) was not controlled for when the independent relationship between cancer incidence rate among the people aged 0–49 years and *I*_s_ was studied because this potential confounder is not relevant to this group of people.

We controlled for GDP not only because it stands for cancer treatment service, but also because it is associated with cancer diagnoses level. Therefore, we considered GDP as a potential confounder and controlled for in our data analysis, which may reduce the influence of GDP associated cancer diagnose rate.

Urbanization, representing a major demographic shift, entails lifestyle changes, including diet with more energy dense components, such as high fat, high alcohol consumption, less vegetables and fruits in daily diet, and less physical exercise (Allender et al., 2008; Moore et al., 2003; WHO, 2010).

Those individual (site) cancers whose incidence rates were significantly and negatively correlated with *I*_s_ in partial correlation are classified as “cancers with strong genetic background.” Those individual cancers whose incidence rates were not significantly or negatively correlated with *I*_s_ are called “Less genetic cancers.”

Cohen's *f* ^2^ was used to calculate and to report the “effect size” in this study.

1. Standard multiple linear regression (enter) was performed to describe the relationships between the outcome variables (all cancers among all ages and 0–49 years age group) and the explanatory variables (GDP, life expectancy [*e*_50_], obesity prevalence rate, physical inactivity prevalence rate, smoking (Danaei et al., 2005) and urbanization (Danaei et al., 2005)). Standard multiple linear regression (stepwise) was performed to identify the most significant predictors of all cancer incidence rates among all ages and 0–49 years, respectively.

Life expectancy of older people (*e*_50_) was not included as an independent predictor in the standard multiple linear regression analysis when we explored the relationships between all cancer incidence rate among the population aged 0–49 years and *I*_s_ because this potential confounder is not relevant to this group of people.

1. To demonstrate the universal association between all cancer incidence rate (all ages) and *I*_s_, we categorized the countries for correlation analyses based on the following: (i) the WHO regional classifications, Africa (AFR), Americas (AMR), Eastern Mediterranean (EMR), Europe (EU), South‐East Asia (SEAR) and Western Pacific (WPR) (2010WHO); (ii) the World Bank income classifications: high income, upper middle income, low‐middle income and low income; and (iii) countries with the strong contrast in terms of geographic distributions, per capita GDP levels and/or cultural backgrounds. We analysed the correlation in the six country groupings: the Arab World (The World Bank, 2015), countries with English as the official language (government websites), the Organisation for Economic Co‐operation and Development (OECD) (The OECD, 2015), the Asia‐Pacific Economic Cooperation (APEC) ([Link]Asia‐Pacific Economic Cooperation), Asia Cooperation Dialogue (ACD) ([Link]Asia Cooperation Dialogue) and the Southern African Development Community (SADC). In our analysis, we only included those countries for which we could access their data for the specific groupings. To a large extent, grouping countries for analysis may also allow us to align our findings against previous local or regional studies regarding heterogeneous cancer epidemiology due to various geographic locations and ethnicity.

Socio‐economic level in different regions has been considered as the major contributor to regional variations of cancer incidence rates (Ferlay et al., 2013; Ferlay et al., 2015; Jemal et al., 2011). Therefore, the correlation coefficients between groupings in different socio‐economic levels were compared with Fisher's *r*‐to‐*z* transformation.

1. IARC‐WHO has reported that GDP is associated with cancer incidence rate (Ferlay et al., 2013, 2015; IARC, 2012; Jemal et al., 2011). Naturally, this drove us to consider the incidence rate of all cancers (all ages) without the contributing effect of GDP. This allows us to explore the association between *I*_s_ and incidence rate of all cancers (all ages) which excludes the contributing effect of GDP.

**Figure 2:**
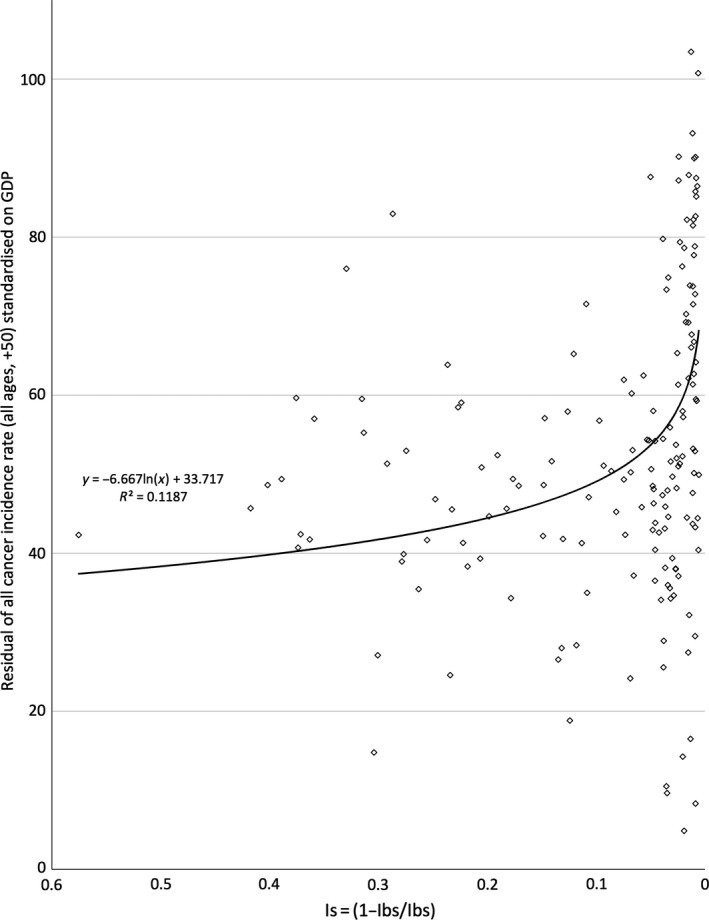
The relationship between *I*_s_ and residual of all cancer incidence rate (+50, all ages) standardized on gross domestic product per capita (GDP)

Scatter plots (simple regression analysis) were used to explore and visualize the correlations between all cancer incidence rate (all ages) and *I*_s_. The strength and form of the relationship between incidence rate of all cancers (all ages) and *I*_s_ was analysed using actual values of the two variables. The equation of the best fitting trendline (logarithmic) displayed in the scatter plots analysis of relationship between GDP and all cancer (all ages) incidence rate was used to calculate and remove the contributing effect of GDP on all cancer (all ages) incidence rate. This allowed us to obtain a new dependent variable “Residual of all cancer (all ages) incidence standardized on GDP.” The relationship between *I*_s_ and “Residual of all cancers (all ages) incidence standardized on GDP” was explored with scatter plots (Figure 2).

In order to assess the magnitude of possible changes in the incidence of cancers due to relaxation of natural selection, we have calculated a “rate of incidence increase” by dividing the average incidence rates in the 10 countries with the lowest *I*_s_ values by the average incidence rates in the 10 countries with the highest *I*_s_ values. These rates allow us to estimate to what extent alteration of the mutation‐selection balance over short periods could be responsible for the change in incidence. This is an approximate measure because other (nongenetic) factors may also influence incidence rates.

Pearson, nonparametric and partial correlations, and the multiple linear regression analysis were conducted using SPSS v. 22 (SPSS Inc., Chicago, IL, USA). Scatter plots and calculation of “Residual of all cancer (all ages) incidence standardized on GDP” were performed in Excel^®^ (Microsoft 2016). The raw data are used for scatter plots and calculation of “Residual of all cancer (all ages) incidence standardized on GDP.” The significance value is recorded for each correlation, and significance level is kept at the 0.05, but 0.01 and 0.001 levels can be found from the reported actual significance values. Standard multiple linear regression analysis criteria were set at probability of *F* to enter ≤.05 and probability of *F* to remove ≥.10.

## 3 RESULTS

The relationship between *I*_s_ and all cancer incidence was negative and strong (*R* ^2^ = 0.5435, Figure 1). When the contributing effect of GDP on all cancer incidence rates was removed, *I*_s_ was still in negative and significant correlation to all cancer incidence (*R* ^2^ = 0.1187, Figure 2).

Globally, *I*_s_ was significantly and negatively correlated with the incidence rates of all cancers at all ages (*r* = −.738, *p* < .001) and at 0–49 years (*r* = −.719, *p* < .001) in Spearman rho analysis (Table 1). This relationship trend remained (*r* = −.319, *p* < .001 and *r* = −.380, *p* < .001, respectively) when we controlled for potential confounding effects of GDP, life expectancy, obesity, physical inactivity, smoking and urbanization in partial correlation analysis (Table 1). When exploring partial correlations of *I*_s_ to individual cancers, significant negative correlation was found for 15 of 27 site cancers (Table 1). Similar results were observed in the correlation analysis with the data comprising 64 countries with “high‐quality” data (Table 1). Rates of incidence increase for all cancers at all ages (2.3) and in 0–49 years age group (2.4) are practically the same, while for individual cancers these rates of incidence increase vary from fractional (=decrease) for cancers not significantly or not negatively correlated with *I*_s_ to over 10 for some cancers significantly negatively correlated with *I*_s_. Overall, for cancers with the strong genetic background which were significantly negatively correlated with *I*_s,_ the average rate of incidence increase is 5.7 while for the less genetic cancers, the average rate of incidence increase is 2.1.

**Table 1:** Spearman rho and partial correlations between *I*_s_ and all cancers incidence rates at all ages and 0‐49 years, and for 27 separate site cancers (Bold indicates significant coefficients)

Independent variables (Cancer)	All countries							Incidence	Countries with “high‐quality” data						
	Nonparametric			Partial^b^					Nonparametric			Partial^b^			
	rho	*p*	*n*	*r*	*p*	*df*	Effect size	Increase^c^	rho	*p*	*n*	*r*	*p*	*df*	Effect size
All cancers (C00‐97, but C44), all ages	−0.738	<.001	173	−**.319**	<.001	98	0.113	2.326	−0.650	<.001	64	−**.348**	.024	40	0.132
All cancers (C00‐97, but C44), 0–49^a^	−0.719	<.001	173	−**.380**	<.001	99	0.168	2.386	−0.607	<.001	64	−**.446**	.003	41	0.248
Bladder (C67)	−0.709	<.001	173	−**.217**	.030	98	0.049	4.126	−0.571	<.001	64	−.248	.114	40	0.065
Brain (C70‐72)	−0.738	<.001	170	−**.247**	.013	98	0.065	6.585	−0.389	<.001	64	−**.405**	.008	40	0.196
Breast (C50)	−0.737	<.001	173	−**.290**	.003	98	0.092	2.698	−0.723	<.001	64	−.300	.054	40	0.099
Cervix uteri (C53)	0.608	<.001	173	.071	.485	98	0.005	0.206	0.407	<.001	64	−.040	.803	40	0.002
Colorectum (C18‐21)	−0.845	<.001	173	−**.455**	<.001	98	0.261	7.008	−0.723	<.001	64	−**.433**	.004	40	0.231
Corpus uteri (C54)	−0.674	<.001	172	−**.337**	<.001	98	0.128	3.837	−0.528	<.001	64	−**.405**	.008	40	0.196
Gallbladder (C23‐24)	−0.509	<.001	158	−**.226**	.024	98	0.054	5.230	−0.096	.452	63	.106	.502	40	0.011
Hodgkin lymphoma (C81)	−0.666	<.001	166	−**.270**	.007	98	0.078	3.314	−0.491	<.001	64	−**.347**	.024	40	0.137
Kaposi sarcoma (C46)	0.564	<.001	120	.325	.004	76	0.118	0.061	0.286	.052	47	.275	.128	30	0.082
Kidney (C64‐66)	−0.850	<.001	167	−**.485**	<.001	98	0.308	10.023	−0.562	<.001	64	−**.425**	.005	40	0.221
Larynx (C32)	−0.448	<.001	168	−.144	.154	98	0.021	2.248	0.005	.966	64	−.182	.248	40	0.034
Leukaemia (C91‐95)	−0.800	<.001	171	−**.392**	<.001	98	0.182	3.574	−0.585	<.001	64	−**.352**	.022	40	0.141
Lip and oral cavity (C00‐08)	−0.257	<.001	173	−.037	.712	98	0.001	1.334	−0.359	.004	64	−**.335**	.030	40	0.126
Liver (C22)	0.300	<.001	173	−.033	.745	98	0.001	0.624	0.041	.750	64	.136	.392	40	0.019
Lung (C33‐34)	−0.782	<.001	173	−**.295**	.003	98	0.095	11.933	−0.483	<.001	64	−.244	.119	40	0.064
Melanoma of skin (C43)	−0.482	<.001	168	−.155	.124	98	0.025	10.286	−0.613	<.001	63	−.283	.069	40	0.087
Multiple myeloma (C88, C90)	−0.663	<.001	157	−**.236**	.018	98	0.059	4.257	−0.547	<.001	64	−.077	.626	40	0.006
Nasopharynx (C11)	0.221	<.001	154	.114	.257	98	0.013	0.767	0.334	.008	63	.144	.364	40	0.021
Non‐Hodgkin lymphoma (C82‐85, C96)	−0.524	<.001	173	.031	.756	98	0.001	2.019	−0.565	<.001	64	−.114	.472	40	0.013
Oesophagus (C15)	0.009	.907	172	.132	.189	98	0.018	0.529	0.008	.951	64	−.004	.978	40	0.000
Other pharynx (C09‐10, C12‐14)	−0.347	<.001	168	−.091	.367	98	0.008	2.037	−0.371	.003	63	−.263	.093	40	0.074
Ovary (C56)	−0.608	<.001	173	−**.309**	.002	98	0.106	1.997	−0.449	<.001	64	−**.469**	.002	40	0.282
Pancreas (C25)	−0.802	<.001	170	−**.453**	<.001	98	0.258	5.142	−0.602	<.001	64	−**.396**	.009	40	0.186
Prostate (C61)	−0.498	<.001	173	−.114	.260	98	0.013	3.463	−0.577	<.001	64	−.301	.053	40	0.100
Stomach (C16)	−0.412	<.001	173	−**.243**	.015	98	0.063	1.826	0.049	.700	64	−.281	.072	40	0.086
Testis (C62)	−0.777	<.001	153	−**.315**	<.001	98	0.110	14.579	−0.681	<.001	64	−**.459**	.002	40	0.267
Thyroid (C73)	−0.684	<.001	170	−**.322**	<.001	98	0.115	5.362	−0.346	.005	64	−.113	.477	40	0.013
GDP PPP 2010	−0.853	<.001	168	–	–	–	–	–	−0.760	<.001	63	–	–	–	–
Life expect (*e*50), 1990–1995	−0.822	<.001	173	–	–	–	–	–	−0.666	<.001	64	–	–	–	–
Obesity	−0.572	<.001	173	–	–	–	–	–	−0.003	.984	64	–	–	–	–
Physical inactivity	−0.315	<.001	132	–	–	–	–	–	−0.103	.453	55	–	–	–	–
Smoking, Daily any tobacco product 2011	−0.551	<.001	123	–	–	–	–	–	−0.234	.086	55	–	–	–	–
Urbanization 2010	−0.712	<.001	169	–	–	–	–	–	−0.455	<.001	64	–	–	–	–

a Life expectancy (*e*50) was not controlled as it is not relevant in people aged 0–49 years.

b Partial correlations were calculated when GDP, life expectancy (*e*50), obesity, physical inactivity, smoking and urbanization were kept statistically constant.

c Ratio of lowest 10 *I*_s_ countries to highest *I*_s_ 10 countries: Average strong genetic cancer incidence rate ratio: 5.718, Average less genetic cancer incidence rate ratio 2.143. “Strong genetic cancer” refers to those individual (site) cancers whose incidence rates were significantly and negatively correlated with *I*_s_ in partial correlation. “Less genetic cancers” refers to those individual cancers whose incidence rates were not significantly or negatively correlated with *I*_s_.

**Figure 3:**
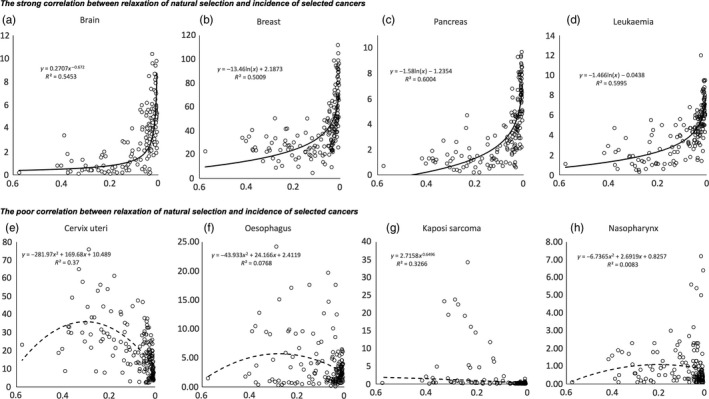
The relationship between relaxation of natural selection and incidence of selected cancers

Relationships between *I*_s_ and some site cancer correlations are illustrated in Figure 3. As can be seen, cancers that had predominantly external causes such as cervical cancer or oesophageal cancer showed no correlation to *I*_s_, while those with possible genetic background do correlate with *I*_s_. Partial correlation between *I*_s_ and 15 cancers remained significant after removal of the confounding effects (Table 1) of the six potential confounders.

The multiple linear regression model (Table 2) showed that, globally, *I*_s_ had the greatest beta coefficient than the potential confounders in the “Enter” analysis, whereas the stepwise regression model identified *I*_s_ as the most significant predictor of all cancers incidence rates among all ages and 0–49. Similar results were revealed after the multiple linear regression model was calculated within the dataset which only included those 64 countries with “high‐quality” data.

**Table 2:** Results of multiple linear regression analyses to identify predictors of cancer incidence

Variable	All countries (*n* = 173)				Variable	Countries with “high‐quality” data (*n* = 64)			
	All ages		0–49 years old			All ages		0–49 years old	
	Beta	Sig.	Beta	Sig.		Beta	Sig.	Beta	Sig.
Enter									
*I*_s_	−0.373	0.014	−0.523	0.000	*I*_s_	−0.809	0.011	−1.090	<0.001
GDP	0.190	0.207	0.254	0.000	GDP	−0.417	0.174	−0.308	0.345
Life expectancy	0.126	0.241	–	–	Life expectancy	0.434	0.009	–	−
Obesity	0.100	0.211	0.040	0.608	Obesity	0.213	0.087	−0.002	0.987
Physical Inactivity	−0.071	0.339	−0.057	0.436	Physical inactivity	−0.232	0.092	−0.192	0.189
Smoking	0.220	0.007	0.204	0.011	Smoking	−0.016	0.911	0.015	0.920
Urbanization	−0.059	0.499	−0.164	0.056	Urbanization	−0.131	0.547	−0.224	0.336

All countries (*n* = 173)					Countries with “high‐quality” data (*n* = 64)				
All ages		0–49 years old		All ages			0–49 years old	
Rank	Variable	Adjusted *R* ^2^	Variable	Adjusted *R* ^2^	Rank	Variable	Adjusted *R* ^2^	Variable	Adjusted *R* ^2^
Stepwise									
1	*I*_s_	0.589	*I*_s_	0.597	1	*I*_s_	0.400	*I*_s_	0.340
2	Smoking	0.616	Smoking	0.625	2	Physical inactivity	0.442	Urbanization	0.442
3	–	–	–	–	3	Life expectancy	0.492	–	–

*I*_s_ was correlated with incidence rate of all cancers (all ages) universally in all country groupings (Table 3). However, there was a tendency for the correlations to be stronger in the more developed country groupings than those in the less developed groupings. This trend was revealed in country groupings divided in consideration of geographic locations (5 WHO regions), income classifications (four groups of the World Bank) and other factors, such as cultural backgrounds (Arab World, countries with English as official language) and international organizations (OECD, APEC, ACD, SADC).

**Table 3:** Associations between *I*_s_ and cancer incidence (all ages) in different country groupings

	Pearson's *r*		Nonparametric	
	Pearson's *r*	Significance	Spearman's rho	Significance
WHO region				
AFRO, *n* = 44	−0.151	0.329	−0.099	0.523
AMRO, *n* = 29	−0.695	<0.001	−0.662	<0.001
EMRO, *n* = 21	−0.043	0.852	0.081	0.729
EURO, *n* = 49	−0.800	<0.001	−0.738	<0.001
SEARO, *n* = 11	−0.034	0.920	−0.191	0.574
WPRO, *n* = 19	−0.590	0.008	−0.599	0.007
The World Bank income				
High, *n* = 48	−0.402	0.005	−0.311	0.032
Upper middle, *n* = 48	−0.647	<0.001	−0.577	<0.001
Low middle, *n* = 47	−0.425	0.003	−0.418	0.003
Low, *n* = 30	−0.166	0.381	−0.104	0.586
Other country groupings				
Arab World, *n* = 21	−0.152	0.512	−0.086	0.710
English, official language, *n* = 50	−0.692	<0.001	−0.625	<0.001
OECD, *n* = 33	−0.539	<0.001	−0.204	0.255
APEC, *n* = 19	−0.616	0.005	−0.730	<0.001
ACD, *n* = 27	−0.447	0.019	−0.373	0.055
SADC, *n* = 14	−0.243	0.403	−0.169	0.563

The more developed regions, Americas and Europe, had stronger correlations than those in other regions. Fisher's *r*‐to‐*z* transformation revealed that the correlation between *I*_s_ and incidence rate of all cancers (all ages) in Europe was significantly stronger than those in the three developing regions, Africa (*z* = 4.41, *p* < .001), Eastern Mediterranean (*z* = 3.8, *p* < .001) and South‐East Asia (*z* = 2.78, *p* = .0027). It was also revealed that in the World Bank income classifications, the correlation between *I*_s_ and incidence rate of all cancers (all ages) in the upper middle income grouping was significantly stronger than that in low‐income classification (*z* = 2.48, *p* = .0066).

The correlation between *I*_s_ and incidence rate of all cancers (all ages) in high‐income classification was not as strong as that in the upper middle classification. It was almost the same as that in the low‐middle classification (Table 3).

## 4 DISCUSSION

Incidence rates of all cancers and of most separate site cancers showed strong and significant correlation to reduced natural selection, measured by *I*_s_. It is especially important that this relationship is strong in the younger part of the population which are in prereproductive (0–14) and reproductive (15–49) periods.

It is important that we list the limitations, including the intrinsic limitations (conceptualized as ecological fallacy) to this study, before examining the public health implications of our results. Firstly, the data included in this study were for whole nations, so we may only demonstrate the relationships between *I*_s_ and cancer incidence at macrolevel. We also need to note that it is nearly impossible to test such relationship at the individual or germline level due to very rare cancer occurrence rates. Secondly, data compiled and/or collected by the major international agencies (WHO, IARC and the World Bank) are fairly crude and may contain some random errors arising from methods of reporting incidence of specific diseases, reliability of diagnoses and possible administrative errors. Thirdly, not all the contributing factors, such as alcohol consumption, can be included as the potential confounders in data analysis due to data availability or quality. Furthermore, the opportunity for natural selection is only measured with respect to postnatal mortality, while gametic selection and intrauterine mortality are not included (Rühli & Henneberg, 2016). Despite these limitations, the findings in this study from different data analysis methods constantly and consistently showed significant correlation between reduced natural selection and all cancers incidence (all ages and 0–49, respectively) and incidence of most of site‐specific cancer groups, especially those for which genetic background may be expected. Obviously, the changes in the genetic code of the human populations may not fully explain the increasing cancer incidence rate. These changes may be cumulative, each one of minor effect and may contribute to increasing cancer incidence together with other carcinogenic factors.

Various genes contribute to cancer, for example proto‐oncogenes, can increase proliferation of mutated cells and tumour suppressor genes could inhibit self‐regulation of abnormal cells, but their balance may still increase cancer incidence in various ways because these genes have pleiotropic effects. In this study, some of cancer groups have incidences that do not correlate with *I*_s_ value, or even show reversed correlations (Table 1, Figure 3). These include cancers of well‐known viral causes—cervical cancer—immune problem‐related cancers like non‐Hodgkin lymphoma, and cancers caused by toxins, like lip and oral cavity cancers. These cancers also have incidence “increases” below zero indicating their greater incidence in countries with larger opportunity for natural selection. This is most likely a result of countries with greater mortality having also poorer hygienic conditions and less medical services.

While specific genes determining risks of specific cancers may be still unknown, the general tendency is clear—relaxation of natural selection allows accumulation of detrimental genetic material, especially if single detrimental alleles have mild effects (Henn et al., 2015). Studies have shown that a partially heritable disease, phenylketonuria, was only noticeable after being accumulated for several generations (Stephan & Henneberg, 2001) with about 2% increase each (Medawar, 1971). Two recent studies also reported that relaxed natural selection has been contributing to the increasing prevalence of two noncommunicable diseases, obesity (Budnik & Henneberg, 2017) and type 1 diabetes (You & Henneberg, 2016) because it may allow detrimental gene accumulation in human population. However crudely calculated our rates of “incidence increase” (Table 1) indicate rates of increase compatible with alteration of mutation‐selection balance. We only have at our disposal recent data, but it can be hypothesized that observed differences among countries in the opportunity for natural selection have existed for a few generations. With a simple accumulation of mutations under zero selection, the incidence rates should double every generation, when selection is not entirely relaxed, but still strongly limited, the increase will be somewhat less than double. Considering that declines in mortality in “developed” countries started in the second half of the 19th century, we can estimate that changes in mutation‐selection rates occurred over lifetime of some four, maybe five, generations. Incidence increases of all cancers (2.3–2.4) indicate approximately doubling over that time, while for cancers correlated significantly with *I*_s_ the average increase is 5.7. Of course, not the entire incidence increase can be attributed to alteration in mutation‐selection balance, because quality of data collection and reporting and presence of carcinogenic external factors may differ between the 10 countries of the lowest opportunity for selection and 10 countries of the highest selection opportunity. Our choice of 10 countries of each kind, instead of only five or 20, also influences precision of the numerical indices calculated. What is important here is that the order of magnitude of incidence increases, and their positive relationship to the relaxation of selection, especially in cancers with supposed genetic background, is compatible with expectations of population genetics. In short—such increases in the incidence of cancers are possible upon significant relaxation of natural selection through differential mortality.

Overall, cancer is an inheritable noncommunicable disease due to its strong genetic background. Cancer genes may be cumulative at the reduced natural selection. Natural selection in the past had an ample opportunity to eliminate defective genes introduced by mutations (Budnik, Liczbińska, & Gumna, 2004; Henneberg, 1976; Henneberg & Henneberg, 1998, 2002; Rühli & Henneberg, 2013, 2016; Saniotis & Henneberg, 2011, 2012; Stephan & Henneberg, 2001). However, natural selection has been significantly reduced in the past 100–150 years, and the direct consequence of this process is that nearly every individual born into a population can pass genes to the next generation, while some 150 years ago, only 50% or less of individuals had this chance (Rühli & Henneberg, 2016; Saniotis & Henneberg, 2012). Therefore, population allowing more people with cancer genes survive reproduction cycle may boost cancer gene accumulation. For instance, genetic predisposition to childhood leukaemia exists (Stieglitz & Loh, 2013). Patients who survive it will have a chance to pass this predisposition to the next generation. Similar argument may be made with respect to other cancers occurring during prereproductive or reproductive period of life. Currently used cancer treatments are not targeting genetic causes of the disease, but dealing with its phenotypic expressions—tumours that are surgically removed, or metastatic cell masses whose proliferation is curtailed by chemotherapy and radiation therapy. Although successful in a portion of cases, these treatments have side effects and do not deal directly with the cause of the disease, therefore, though undoubtedly helpful to a number of patients, they are not optimally effective.

Table 3 showed that country groupings with higher socio‐economic level had stronger associations between *I*_s_ and cancer incidence. This finding is consistent with the studies conducted by the WHO cancer research agent, IARC (Ferlay et al., 2010, 2013, 2015; Jemal et al., 2011). Similarly, reduced natural selection and type 1 diabetes prevalence also showed stronger association in developed regions (You & Henneberg, 2016). One of the explanations may be that people in developed regions, such as Europe and America have been able to access better health services, which has made them to escape natural selection more often and pass their detrimental genes onto their next generation. The long effect from escaping natural selection may allow those genes, including cancer‐related genes, to accumulate in those populations faster (Medawar, 1971; Stephan & Henneberg, 2001; You & Henneberg, 2016).

The association between *I*_s_ and cancer incidence was strong and significant in both upper middle and high‐income economic classifications (the World Bank). However, it was stronger in upper middle income economic classification. This may be attributable to the fact that almost all people in the countries in high‐income grouping may be able to escape natural selection due to high level of health services. This is shown by the extremely low *I*_s_ values of these countries, which are close to 0 (Data S1). (ii) Fast developing GDP in upper middle country grouping has driven their medicine level to develop quickly, which may have made more and more people escape natural selection.

## 5 CONCLUSION

Assuming that the increasing genetic load underlies cancer incidence as one of the contributing factors, the only way to reduce it remains genetic engineering—repair of defective portions of the DNA or their blockage by methylation and similar approaches. These techniques, though theoretically possible, are not yet practically available. They will, however, need to be developed as they provide the only human‐made alternative to the disappearing action of natural selection as any eugenics‐like approaches are ethically and morally reprehensible.

## DATA ARCHIVING STATEMENT

All data for this study are publicly available and are ready for the public to download at no cost from the official websites of the World Bank, the WHO and FAO. Use of these data for this research falls within this UN agency's public permission in their terms and conditions. There is no need to have the formal permission to use the data for this study. We pooled those data into the Excel and attached to our manuscript as the supplemental document.
